# The COVID-19 Pandemic Increased the Incidence of New-Onset Type One Diabetes in Children

**DOI:** 10.3390/children11020142

**Published:** 2024-01-23

**Authors:** Orit Blumenfeld, Mikhail Rozenshmidt, Idan Eini, Zvi Laron

**Affiliations:** 1 Israel Center for Disease Control, Ministry of Health, Ramat-Gan 5262000, Israel; mikhail.rozenshmidt@moh.gov.il (M.R.); idan.eini@moh.gov.il (I.E.); 2 Endocrinology and Diabetes Research Unit, Schneider Children’s Medical Center, Petah Tikva 4920235, Israel; laronz@clalit.org.il

**Keywords:** type 1 diabetes, COVID-19, incidence, children, public health, epidemiology

## Abstract

**Background**: The impact of the COVID-19 pandemic on the incidence rate of childhood type 1 diabetes (T1D) is controversial. Our aim was to analyze the incidence of new-onset T1D among children aged 0–17 before and during the COVID-19 pandemic in Israel. **Methods**: Data obtained from the national T1D registry for children aged 0–17 were analyzed for the pre-pandemic (1997–2019) and pandemic (2020–2022) periods. In the pre-pandemic period, 7246 children with newly diagnosed T1D were compared with 1490 children diagnosed during the pandemic period. **Results**: T1D incidence significantly increased in the 0–17 age group from a mean of 12.9/10^5^ (pre-pandemic) to 17.7/10^5^ and 16.7/10^5^ during the first two years of the pandemic (2020 and 2021, respectively) (*p* = 0.0001). Stratifying by age group (0–4, 5–9, 10–14, and 15–17) revealed a significant increase in the 5–9, 10–14, and 15–17 groups, both in 2020 (*p* = 0.0001) and in 2021 (*p* = 0.0001). The incidence rate in the 0–4 age group showed no change in the first year of the pandemic (2020) (*p* = 0.4). However, in the second year of the pandemic (2021), there was a significant increase from 6.3/10^5^ in the pre-pandemic period to 9.1/10^5^ (*p* = 0.001). Anti-COVID-19 vaccination in 2022 led to a significant decrease in the incidence rates in the 10–14 and 15–17 age groups (*p* = 0.03 and *p* = 0.02, respectively). **Conclusion**: The COVID-19 pandemic was associated with a significant increase in the incidence of new-onset T1D in prepubertal and pubertal children. Anti-COVID-19 vaccination decreased the incidence rate significantly only in pubertal children.

## 1. Introduction

Type 1 diabetes mellitus (T1D) is an autoimmune disease caused by the gradual destruction of the pancreatic insulin-producing β-cells [1]. The ongoing global rise in the prevalence of childhood T1D [2] is believed to be driven by the increasing frequency of viral infections [3,4]. Among the most commonly implicated viruses are enteroviruses, such as Coxsackie B4 [4,5,6]. In recent years, a connection between perinatal rotavirus infections and T1D has been established [7,8,9,10]. Previous data had demonstrated that the rubella [11] and mumps viruses [12] were responsible for early-onset diabetes in children, and vaccinations against these two viruses have effectively eliminated this causative link. In December 2019, a novel coronavirus SARS-CoV-2 infection, also known as the COVID-19 pandemic, spread to most countries in the world, leading to extreme public health measures, including widespread lockdowns [13]. It has been hypothesized that, like the SARS-CoV-1 virus, the SARS-CoV-2 virus also binds to the ACE2 receptor, which is widely expressed in pancreatic cells. The hypothesis suggests that the virus penetrates through the ACE2 receptor into the beta cells of the pancreas, causing their destruction, which leads to the occurrence of T1DM [14,15].

Information regarding the impact of SARS-CoV-2 infection on pancreatic β-cells remains limited. Research on whether SARS-CoV-2 infects pancreatic β-cells has yielded inconclusive results [16,17,18], and data on the incidence of newly diagnosed childhood T1D during the COVID-19 pandemic are scarce [19,20].

In late February 2020, the COVID-19 pandemic reached Israel. From 1 March 2020 to 22 December 2020, the country experienced the pandemic before the introduction of the BioNTech-Pfizer mRNA-BNT-162b2 anti-COVID-19 vaccine to its population. The vaccine was introduced on 23 December 2020 for ages 12 years and above, and from October 2021 for ages 5 years and above. From June 2022, two doses of vaccination became available for all age groups.

In children, COVID-19 can be difficult to diagnose, as the presentation is often subclinical and the children are asymptomatic [21]. One study showed that 86% of children infected with the virus in China at the beginning of the epidemic were not diagnosed [22]. The rate of diagnosed children varies between 2% in China and 5% in the USA [23,24,25]. It is possible that children exposed to a confirmed patient may also be at risk of developing T1DM. This is the reason we did not merge our national new-onset childhood T1D registry with the COVID-19 morbidity national database.

The aim of the current study was to investigate the incidence of newly diagnosed T1D before and during the COVID-19 pandemic for the 0–17 age group in Israel by using only our national new-onset childhood T1D registry.

## 2. Materials and Methods

Data were retrieved from the national childhood T1D registry, which covers the entire country, and from the national COVID-19 morbidity and vaccination database. The information on T1D was collected from 20 diabetes clinics submitted to the Israel Center for Disease Control (ICDC) since 1997. A comparison was made between the mean incidence rates during the pre-pandemic period (1997–2019) and the COVID-19 pandemic period (2020–2022). Additionally, comparisons were made between the pre-vaccination year (2020), the year of vaccination introduction (2021), and the year when vaccines became available to all ages (2022). The study was approved by the Israel MOH Supreme Review Board (COR-MOH-097-2021) in November 2021.

### Statistical Analysis

Categorical data were expressed as absolute numbers with percentages. Continuous variables were expressed as means and standard deviations (SDs). Annual rates for T1D were referenced to the overall population of Israel for each year between 1997 and 2022 available from the Israeli Central Bureau of Statistics. Statistical analysis was performed using the SAS package (version 9.4, SAS, Cary, NC, USA). *p* < 0.05 was considered statistically significant for all analyses.

## 3. Results

Table 1 summarizes the results of the study for the entire population aged 0–17 years, segregated by sex and age group. A comparison was made between the 7246 children (3427 girls and 3819 boys) diagnosed during the pre-pandemic period and 1490 children (691 girls and 799 boys) diagnosed during the pandemic years (2020–2022). During the first year of the COVID-19 pandemic (2020), the overall incidence rose significantly from 12.9/10^5^ to 17.7/10^5^ (*p* = 0.0001) followed by an increase to 16.7/10^5^ in the second year (*p* = 0.0001). The rise was consistent in both girls and boys (*p* = 0.0001).

A subsequent analysis comparing the pre-vaccination period (2020) with the year when vaccination became available to all ages (2022) revealed a decrease in incidence rates from 17.7/10^5^ to 14.1/10^5^, respectively (*p* = 0.0005).

When dividing the study population by age group, variations in incidence rates were observed. The 0–4 age group showed no significant increase in incidence in the first year of the pandemic (2020) when compared to the pre-pandemic period (*p* = 0.4). During the second year of the pandemic (2021) this group had a significant increase from 6.3/10^5^ (pre-pandemic period) to 9.1/10^5^, (*p* = 0.01).

In the 5–9 age group, the incidence rate increased significantly from 14.4/10^5^ (pre-pandemic period) to 18.5/10^5^ in 2020, the first year of the pandemic (*p* = 0.002), and further to 19.0/10^5^ in 2021, the second year of the pandemic (*p* = 0.001). In the 10–14 age group, the incidence rate showed a significant rise from 19.3/10^5^ (pre-pandemic) to 27.6/10^5^ in 2020 (*p* = 0.0001) and then to 23.3/10^5^ in 2021 (*p* = 0.01). Finally, for the 15–17 age group, the incidence rate rose significantly from 12.0/10^5^ (pre-pandemic) to 20.0/10^5^ in 2020, the first year of the pandemic (*p* = 0.0001), and subsequently to 15.6/10^5^ in 2021, in the second year of the pandemic (*p* = 0.03). An additional comparison was made between the incidence rate before and during the vaccination periods. It became evident that vaccination caused a significant decrease in the incidence rates in the 10–14 and 15–17 age groups, dropping from 27.6/10^5^ to 22.2/10^5^, (*p* = 0.03) in the 10–14 age group and from 20.0/10^5^ to 13.8/10^5^, (*p* = 0.02) in the 15–17 age group. Notably, vaccination did not influence the incidence rate in the 0–4 and 5–9 age groups when comparing 2020 (pre-vaccination) with 2021 (vaccination introduction) (*p* = 0.1 and *p* = 0.8, respectively) even when comparing 2020 (pre-vaccination) with 2022 (vaccination available for all ages) (*p* = 0.3 and *p* = 0.1, respectively).

As shown in Table 2 the highest percentage rate of new-onset cases of T1D occurred during winter both in the pre-pandemic and pandemic periods. On the other hand, summer had the lowest percentage rate both during the pre-pandemic period and the pandemic period. This pattern of new-onset cases was not changed by the pandemic or vaccination. However, when analyzing seasonality by age group, pubertal children in the 10–14 age group exhibited a significant increase in the percentage mean rate (from 24.3% in the pre-pandemic period to 32.1% in the second year of the pandemic (2021), *p* = 0.02, both in the springs season (Table 2)).

Furthermore, in Table 2 it may be observed that the introduction of vaccination in 2021 resulted in a significant decrease in the percentage rate during spring for both the 10–14 age group (*p* = 0.02) and the 15–17 age group (*p* = 0.03), in comparison to the pre-vaccination year (2020) (Table 2).

**Figure 1:**
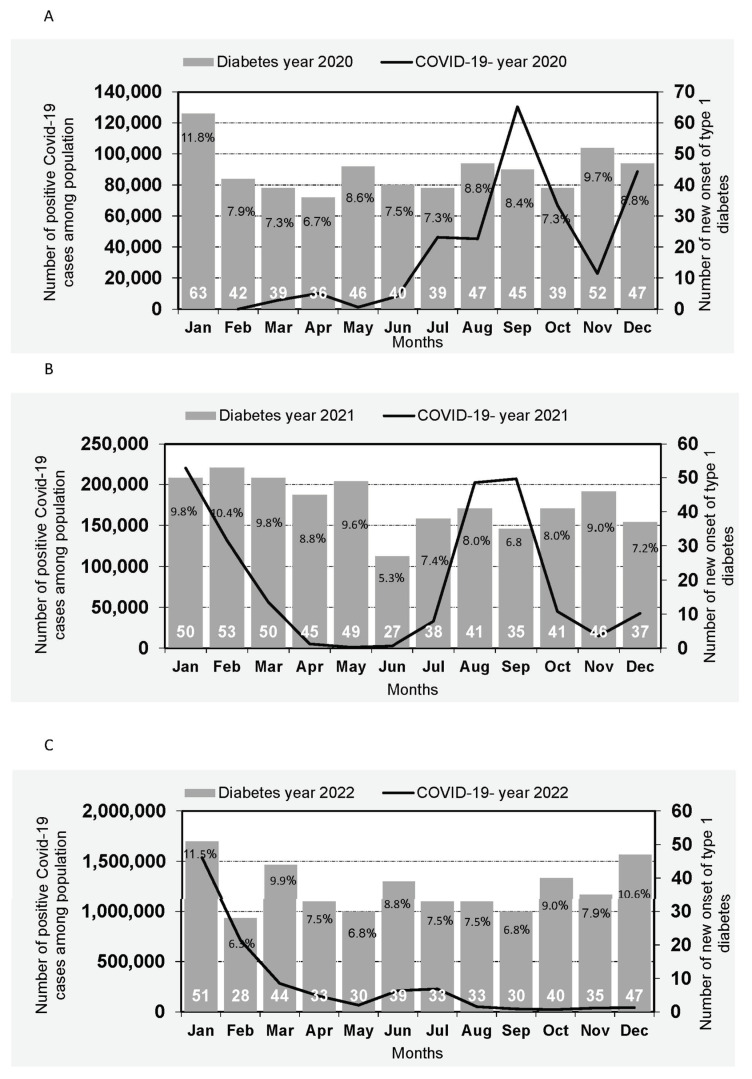
Incidence of type 1 diabetes among Israeli children by Month of diagnosis and incidence of COVID-19 in Israel. (**A**): Number of new onset of type 1 diabetes cases of Israeli children by Month of diagnosis in year 2020 and trend of COVID-19 in Israel by month of the year 2020. (**B**): Number of new onset of type 1 diabetes cases of Israeli children by Month of diagnosis in year 2021 and trend of COVID-19 in Israel by month of the year 2021. (**C**): Number of new onset of type 1 diabetes cases of Israeli children by Month of diagnosis in year 2022 and trend of COVID-19 in Israel by month of the year 2022.

The effect of the waves of the pandemic on the incidence rate is illustrated in Figure 1. Figure 1A illustrates the association between the number of positive COVID-19 cases in Israel during 2020, by month, and the incidence of new-onset T1D among Israeli children in the same year. Similarly, Figure 1B,C depict the corresponding associations for the years 2021 and 2022, respectively. The interaction between waves and the percentage rate of new-onset T1D revealed an increase occurring two months after the peak of a COVID-19 wave among children aged 0–17. In 2020 (Figure 1A), higher rates were observed in May following the March wave and in November following the September wave. In 2021 (Figure 1B), elevated rates were noted in February 2021 after the December 2020 wave and in November 2021 after the September 2021 wave. In 2022, a higher rate of new-onset T1D in children was observed in March 2022 after the January 2022 wave (Figure 1C).

## 4. Discussion

The current study examined the incidence rate of childhood-onset Type 1 diabetes (T1D) before and during the COVID-19 pandemic in Israel. The findings reveal a significant increase during the first two years of the pandemic (2020–2021) among girls and boys aged 5–17, followed by a significant decrease when vaccination became available to all ages in 2022, specifically in the 10–17 age group. According to the data from the national Israeli COVID-19 vaccination database, 74% of children in the 15–17 age group were vaccinated by the end of 2022, and 43.5% of those in the 10–14 age group. During the same period, 81% of the population aged 18 and above had been vaccinated. It is of interest that despite the absence of vaccination, COVID-19 had a lesser effect on the incidence rate of T1D among children aged 0–4 in the first year of the pandemic (2020). There are several possible explanations for these findings: (a) the COVID-19 virus is not the first and acute trigger to destroy 80% of pancreatic β-cells causing T1D. (b) The attenuation of the incidence of T1D in the 0–4 age group induced by the introduction of nationwide rotavirus vaccination in Israel in 2011 [9,10] does not seem to have been affected by the COVID-19 pandemic. (c) The increase in the incidence rate of T1D during the COVID-19 pandemic being limited to the prepubertal and pubertal groups can be attributed to preexisting damage to β-cells. The COVID-19 infection acted as the last hit causing the transition from pre-clinical to clinical diabetes. (d) An additional contributing factor to the higher incidence rate of T1D among children aged 5–17 in the pandemic years 2020 and 2021 may be the stress resulting from the pandemic itself, along with social isolation and school closures [26,27,28].

The findings in our study are supported by a number of publications from other countries. The majority of studies were conducted in single or multiple clinics [29,30,31,32,33,34,35]. Only a few nationwide studies have been carried out, including those in Germany [36], Finland [37], Denmark [38], Romania [19], USA [20], and Scotland [30]. Kamrath C et al. [36] from Germany conducted a national study covering the period from 1 January 2020 to 30 June 2021, involving children aged < 18 years, and reported an increase in the incidence rate of T1D during the COVID-19 pandemic in children aged 0–11 years but not children aged 12–17 years. Salmi H et al. [37] from Finland found in their national study an increase in the incidence rate of new-onset T1D from 2.89/100,000 person-years (PY) in 2016–2019 to 9.35/100,000 PY in 2020, with an incidence rate ratio (IRR) of 3.24 (95% CI 1.80 to 5.83); *p* = 0.001. Zareini B et al. [38] from Denmark found in their national study that the incidence of T1D among individuals aged less than 30 years increased in the April–June 2021 period compared with the same months in the period 2015–2019. Vlad A et al. [19] from Romania utilized a national cohort of children aged 0–14. The findings showed an increase in the incidence of T1D in the first year of the COVID-19 pandemic, rising from 11.4/100,000 in 2019 to 13.3/100,000 in 2020. Qeadan F et al. [20], based on a national US cohort from 1 December 2019 to 31 July 2021, identified a strong association between COVID-19 and the incidence of T1D. Makeigue PM et al. [30] from Scotland used a national registry for the period between March 2020 and November 2021, focusing on individuals aged < 35 years. The study found that among children aged 0–14 years, the incidence rate of childhood T1D during 2020–2021 was 20% higher than in the pre-epidemic period. Two studies from Saudi Arabia and Italy [39,40] based on small populations showed no correlation between the COVID-19 pandemic and the incidence of childhood T1D.

Correlating the waves of the COVID-19 pandemic with a diagnosis of clinical childhood T1D varies between reports. In our study, we observed that the incidence rate was higher two months after the peak of waves. Similar to our findings, Kamrath C et al. [36] found that the peak incidence of T1D occurred three months after the peak of COVID-19. Contrary to these findings, Makeigue PM et al. [30] reported that the rate ratio for T1D incidence associated with a first positive test for SARS-CoV-2 was 2.62 (95%CI 1.81–3.78) for infection in the previous 30 days to a diagnosis of T1D. The progression from infection to clinical T1D could be attributed to the cytokine storm seen in children after COVID-19, indicating a different course of the multisystem inflammatory syndrome (MIS-C) with a focus on autoimmunity [18,36,41].

Higher incidence during the winter and fall periods was probably the result of a higher infection rate during cold weather. Our study confirms previous study findings [42,43] that children were diagnosed with T1D more often in the cold seasons, with 53% of cases diagnosed in fall or winter (*p* = 0.0001). Studies during the COVID-19 pandemic [44] revealed that cold and winter conditions were associated with greater SARS-CoV-2 transmission in Western countries. The drop in temperature and decrease in humidity seem to contribute to the intensification of the spread of virus infections.

The strength of our study is the population-based national T1D registry for children aged 0–17 and the national databases of COVID-19 morbidity and vaccination from Israel. Furthermore, the study covered the entire period of the pandemic (years 2020–2022). Additionally, Israel has a public healthcare system accessible to all citizens; therefore, vaccination was available early in the second year of the pandemic (January 2021).

The limitations of our study include the fact that a high percentage of children who became infected with COVID-19 did not have a COVID-19 test; therefore, there is no information about the number of children who were diagnosed with COVID-19 and T1D.

The results of our study and similar studies from other countries are of paramount importance for shaping healthcare policies, devising pandemic management strategies, and providing public health guidance.

## 5. Conclusions

A comparison between the pre-pandemic period (1997–2019) and the pandemic period (2020–2022) revealed that COVID-19 increased the incidence rate of childhood T1D, especially in the 5–9, 10–14, and 15–17 age groups. Comparison between 2020 (pre-vaccination year), 2021 (introduction of vaccination), and 2022 (vaccination available to all ages) found that anti-COVID-19 vaccination decreased the incidence rate of T1D in children in the 10–14 and 15–17 age groups. The 0–4 age group did not show an increase in incidence rate in 2020—the first year of the pandemic. No clear causal link between COVID-19 and T1D can be drawn from the present study and current literature.

## Figures and Tables

**Table 1:** Changes in the incidence rates of new-onset childhood type 1 diabetes mellitus (T1D) among children aged 0–17 before the COVID-19 pandemic and during the pandemic—before and after anti-COVID-19 vaccination.

	* 2019–1997 (1)			** 2020 (2)			*** 2021 (3)			**** 2022 (4)			P (1 vs. 2)	P (1 vs. 3)	P (1 vs. 4)	P (2 vs. 3)	P (2 vs. 4)	P (3 vs. 4)
	N	Rate	95% CI	N	Rate	95% CI	N	Rate	95% CI	N	Rate	95% CI						
Total annual incidence (ages 0–17)	7246	**12.9/10^5^**	12.6–13.2	535	**17.7/10^5^**	16.2–19.2	512	**16.7/10^5^**	15.2–18.1	443	**14.1/10^5^**	12.8–15.5	**0.0001**	**0.0001**	0.2	0.4	**0.0005**	**0.01**
Girls	3427	**12.5/10^5^**	12.1–12.9	258	**17.5/10^5^**	15.3–19.6	220	**14.7/10^5^**	12.8–16.7	213	**14.0/10^5^**	12.1–15.8	**0.0001**	0.06	0.3	0.06	**0.02**	0.6
Boys	3819	**13.2/10^5^**	12.8–13.6	277	**17.8/10^5^**	15.9–20.1	292	**18.5/10^5^**	16.5–20.7	230	**14.6/10^5^**	12.7–16.5	**0.0001**	**0.0001**	0.2	0.6	**0.01**	**0.003**
Ages 0–4	1096	**6.3/10^5^**	4.5–8.1	65	**7.1/10^5^**	5.4–8.8	83	**9.1/10^5^**	7.1–11.0	54	**15.8/10^5^**	4.2–7.3	0.4	**0.001**	0.5	0.1	0.3	**0.009**
Ages 5–9	2308	**14.4/10^5^**	11.6–17.3	162	**18.5/10^5^**	15.6–21.3	169	**19.0/10^5^**	16.1–21.8	141	**15.5/10^5^**	13.0–18.1	**0.002**	**0.001**	0.4	0.8	0.1	0.08
Ages 10–14	2844	**19.3/10^5^**	15.9–22.7	219	**27.6/10^5^**	23.9–31.2	190	**23.3/10^5^**	20.0–26.6	185	**22.2/10^5^**	19.0–25.4	**0.0001**	**0.01**	0.06	0.08	**0.03**	0.5
Ages 15–17	998	**12.0/10^5^**	8.4–15.5	89	**20.0/10^5^**	15.9–24.2	70	**15.6/10^5^**	11.9–19.3	63	**13.8/10^5^**	10.4–17.2	**0.0001**	**0.03**	0.3	0.1	**0.02**	0.5

* Period 1997–2019 (1)—before the COVID-19 pandemic period; ** 2020 (2)—the first year of the COVID-19 pandemic, pre-anti-COVID-19 vaccination year; *** 2021 (3)—the second year of the COVID-19 pandemic, introduction of anti-COVID-19 vaccination, available nationally to children aged 12+ years (until October 2021) including those aged 5+ years (from October 2021); **** 2022 (4)—the third year of COVID-19 pandemic, vaccination available to all ages nationally (from June 2022). The numbers in bold are the significant *p* value of statistical analysis.

**Table 2:** Incidence of type 1 diabetes by seasons. Comparison among the pre-pandemic period (1997–2019), pandemic period (2020–2022), pre-anti-COVID-19 vaccination year (2020), the introduction of anti-COVID-19 vaccination year (2021), and vaccination available to people of all ages year (2022).

	* 1997–2019 (1)		** 2020 (2)		*** 2021 (3)		**** 2022 (4)		P 1 vs. 2	P 1 vs. 3	P 1 vs. 4	P 2 vs. 3	P 2 vs. 4	P 3 vs. 4
	N	%	N	%	N	%	N	%						
**Seasons (ages 0–17)**														
Winter (N,%)	2048	28.3	152	28.4	140	27.3	126	28.4	0.1	0.7	0.9	0.7	1	0.7
Spring (N,%)	1779	24.6	121	22.6	144	28.1	107	24.2	0.3	0.07	0.9	0.05	0.6	0.2
Summer (N,%)	1596	22	126	23.6	106	20.7	105	23.7	0.4	0.5	0.4	0.3	1	0.3
Autumn (N,%)	1823	25.2	136	25.4	122	23.8	105	23.7	0.9	0.5	0.5	0.6	0.6	1
**Age group by seasons**														
**Age group 0–4**														
Winter (N,%)	287	26.2	19	29.2	25	30.1	19	35.2	0.6	0.4	0.1	0.9	0.5	0.5
Spring (N,%)	289	26.4	19	29.2	19	22.9	10	18.5	0.6	0.5	0.2	0.4	0.2	0.5
Summer (N,%)	237	21.6	15	23.1	23	27.7	12	22.2	0.8	0.2	0.9	0.5	0.9	0.5
Autumn (N,%)	283	25.8	12	18.5	16	19.3	13	24.1	0.2	0.2	0.8	0.9	0.5	0.5
**Age group 5–9**														
Winter (N,%)	659	28.6	47	29	43	25.4	47	33.3	0.9	0.4	0.2	0.5	0.4	0.1
Spring (N,%)	583	25.3	40	24.7	43	25.4	29	20.6	0.9	0.9	0.2	0.9	0.4	0.3
Summer (N,%)	530	23	37	22.8	40	23.7	37	26.2	0.9	0.8	0.4	0.9	0.5	0.6
Autumn (N,%)	536	23.2	38	23.5	43	25.4	28	19.9	0.9	0.5	0.4	0.7	0.4	0.2
**Age group 10–14**														
Winter (N,%)	810	28.5	62	28.3	51	26.8	43	23.2	0.9	0.7	0.1	0.7	0.2	0.4
Spring (N,%)	690	24.3	48	21.9	61	32.1	52	28.1	0.4	**0.02**	0.2	**0.02**	0.2	0.4
Summer (N,%)	616	21.7	52	23.7	33	17.4	46	24.9	0.5	0.2	0.3	0.1	0.8	0.07
Autumn (N,%)	728	25.6	57	26	45	23.7	44	23.8	0.9	0.6	0.6	0.6	0.6	0.9
**Age group 15–17**														
Winter (N,%)	292	29.3	24	27	21	30	17	27	0.6	0.9	0.7	0.7	0.9	0.7
Spring (N,%)	217	21.7	14	15.7	21	30	16	25.4	0.2	0.1	0.5	**0.03**	0.1	0.6
Summer (N,%)	213	21.3	22	24.7	10	14.3	10	15.9	0.5	0.2	0.3	0.1	0.2	0.8
Autumn (N,%)	276	27.7	29	32.6	18	25.7	20	31.8	0.3	0.7	0.5	0.3	0.9	0.4

* Period 1997–2019 (1)—before the COVID-19 pandemic period; ** 2020 (2)—the first year of the COVID-19 pandemic, pre-anti-COVID-19 vaccination year; *** 2021 (3)—the second year of the COVID-19 pandemic, the introduction of anti-COVID-19 vaccination, available nationally to children aged 12+ years (until October 2021) including those aged 5+ years (from October 2021); **** 2022 (4)—the third year of the COVID-19 pandemic, vaccination available to all ages nationally (from June 2022). The numbers in bold are the significant *p* value of statistical analysis.

## Data Availability

Data available on request due to restrictions privacy.
